# Voice Assistant Reminders and the Latency of Scheduled Medication Use in Older Adults With Pain: Descriptive Feasibility Study

**DOI:** 10.2196/26361

**Published:** 2021-09-28

**Authors:** Marcia Shade, Kyle Rector, Kevin Kupzyk

**Affiliations:** 1 College of Nursing University of Nebraska Medical Center Omaha, NE United States; 2 Department of Computer Science University of Iowa Iowa City, IA United States

**Keywords:** adherence, pain medications, older adults, reminders, mHealth, voice assistants

## Abstract

**Background:**

Pain is difficult to manage in older adults. It has been recommended that pain management in older adults should include both nonpharmacologic and pharmacologic strategies. Unfortunately, nonadherence to pain medication is more prevalent than nonadherence to any other chronic disease treatment. Technology-based reminders have some benefit for medication adherence, but adherence behavior outcomes have mostly been verified by self-reports.

**Objective:**

We aimed to describe objective medication adherence and the latency of medication use after a voice assistant reminder prompted participants to take pain medications for chronic pain.

**Methods:**

A total of 15 older adults created a voice assistant reminder for taking scheduled pain medications. A subsample of 5 participants were randomly selected to participate in a feasibility study, in which a medication event monitoring system for pain medications was used to validate medication adherence as a health outcome. Data on the subsample’s self-assessed pain intensity, pain interference, concerns and necessity beliefs about pain medications, self-confidence in managing pain, and medication implementation adherence were analyzed.

**Results:**

In the 5 participants who used the medication event monitoring system, the overall latency between voice assistant reminder deployment and the medication event (ie, medication bottle cap opening) was 55 minutes. The absolute latency (before or after the reminder) varied among the participants. The shortest average time taken to open the cap after the reminder was 17 minutes, and the longest was 4.5 hours. Of the 168 voice assistant reminders for scheduled pain medications, 25 (14.6%) resulted in the opening of MEMS caps within 5 minutes of the reminder, and 107 (63.7%) resulted in the opening of MEMS caps within 30 minutes of the reminder.

**Conclusions:**

Voice assistant reminders may help cue patients to take scheduled medications, but the timing of medication use may vary. The timing of medication use may influence treatment effectiveness. Tracking the absolute latency time of medication use may be a helpful method for assessing medication adherence. Medication event monitoring may provide additional insight into medication implementation adherence during the implementation of mobile health interventions.

## Introduction

Pain is a symptom that is commonly reported by older populations and is a significant problem, as the size of the older adult population is anticipated to reach 98 million by the year 2060 [1,2]. Geriatric pain experts have recommended that older adults should manage pain symptoms via a combination of nonpharmacologic and pharmacologic interventions [3-5]. However, adherence is a problem; on average, 50% of medications prescribed for chronic conditions are not taken by patients. Medication adherence is defined as the process by which patients take medications as prescribed [6]. The concept of medication adherence is delineated into the following process components: initiation, implementation, and discontinuation. One can measure the implementation component of medication adherence by observing the extent to which an individual’s dosing corresponds to the health care provider–prescribed regimen [6]. Nonadherent behaviors in older adults, such as missing medications, taking medications late, and taking different doses, can increase the risk of adverse events, and such events may vary based on medication class [3,7,8].

Medication adherence is important in older patients with pain symptoms. Acetaminophen, nonsteroidal anti-inflammatories, gabapentoids, and opioids are commonly prescribed as analgesic treatments. The use of these medications includes risks that may outweigh these medications’ benefits; these risks are the result of age-related changes in the drug clearance processes of the body [3,7,8]. Medications for analgesia should be initiated at low doses and used for a short duration, and patients’ adherence to such medications should be monitored closely [3-5,7]. Nonadherence to pain medication has been reported to be more prevalent than nonadherence to any other treatment for chronic pathologies [9]. Of those aged 65 years and older, 57% have reported not adhering to medications for pain relief. Nonadherence behaviors have been reported to be a result of a variety of attitudes toward and concerns about pain medications, such as fears of analgesic use and addiction [3,10-12]. Older individuals may also overuse and underuse medications for pain relief, and these behaviors are also considered to be nonadherence behaviors [10,11]. Pain medication adherence needs to be monitored by patients and health care providers to ensure that optimal pain management and the aversion of adverse events occurs among older adults.

Health care providers need to promote self-monitoring and medication adherence as important elements of pain self-management [13]. A supportive strategy for older adults is the use of reminders [14]. Several studies have found that mobile health (mHealth) reminders from smartphones, tablets, and text messages encourage adherence behaviors [15,16]. mHealth interventions may result in desirable health outcomes, but more studies need to be conducted [17,18].

The success of reminder use has been variable, and the analysis of reminder use is based on a research methodology that relies on self-reported adherence as the primary factor for the validation of medication use [19-23]. Self-reported adherence can be an overestimated and biased account of medication adherence [24]. mHealth reminders may be an intervention that promotes adherence behavior, but researchers have not objectively measured the exact timing of medication use. Medication implementation adherence is a health outcome that can be measured over a defined interval of time, such as the number of doses taken on time, in relation to a prescription-defined time interval [6]. The timing of medication dosages can impact the therapeutic effects that treatments have on chronic diseases and symptoms, such as pain. Studies that use reminder-based interventions need to include instruments for capturing implementation adherence.

Voice-controlled assistants are new technologies that extend the accessibility of mHealth apps and have the potential to be extensively used in health care [25]. We conducted a usability study to describe older adults’ use of voice assistant reminders for pain self-management tasks [26]. The aims of this study were to test the feasibility of an objective measure of medication adherence and to describe older adults’ implementation adherence to scheduled pain medication as a health outcome after the use of a voice assistant reminder intervention.

## Methods

This study enrolled a convenience sample of 15 rural and urban community–dwelling adults aged 55 years and older in the Midwest. Adults were eligible if they lacked cognitive impairments, lived independently, had self-reported pain, used scheduled medications for pain relief, and had never used a voice assistant [26]. A subsample of 5 participants were randomly selected (via a random number generator) to use a medication event monitoring system for their scheduled pain medications and a voice reminder over a 4-week duration.

We analyzed the descriptive data that were collected from the participants in order to obtain background information on the subsample. These data included demographics, pain intensity, pain interference, concerns and necessity beliefs about pain medications, and participants’ confidence in self-managing their pain symptoms. The Brief Pain Inventory-Short Form 8A measures pain intensity and pain interference on a 10-point Likert scale. Higher scores indicate higher pain intensity and pain interference. In this study, average scores for pain intensity and pain interference were categorized as mild pain (score: range 1-3), moderate pain (score: range 4-6), and severe pain (score: range 7-10) [27]. The Beliefs About Medicines Questionnaire measures each respondent’s concerns and necessity beliefs about pain medications. The specific necessity and specific concern scales of the Beliefs About Medicines Questionnaire have 5 items with responses that range from strongly disagree to strongly agree and scores that range from 5 to 25; higher scores indicate stronger beliefs [28]. The Patient-Reported Outcomes Measurement Information System (PROMIS) Self-Efficacy for Managing Symptoms Questionnaire measures an individual’s confidence in self-managing their pain symptoms. This questionnaire includes 8 questions with responses on a 5-point Likert scale. In this questionnaire, mean T-scores of >50 are indicative of higher-than-average self-efficacy, and mean T-scores of <50 are indicative of lower-than-average self-efficacy [29].

Documented data on scheduled pain medication names, doses, and frequencies were recorded from participants’ medication bottles. The medication event monitoring of medication implementation adherence was conducted with the MEMS Smart Cap (AARDEX Group). The MEMS cap includes an electronic chip that digitally records the time when pill bottles are opened and thus indirectly measures the time when a medication is being dispensed [30]. The participants’ Google Assistant profiles were reviewed to ensure that daily reminders for the scheduled medication doses were executed on time.

Data were analyzed to describe the characteristics of the subsample. Descriptive statistics analyses were performed on the questionnaires; voice assistant execution times; and MEMS cap medication adherence percentages, which were calculated with the Med Amigo software (AARDEX Group). The absolute measure of latency treated taking medications before the reminder in the same way as it treated taking medications after the reminder. SPSS version 25 (IBM Corporation) was used to perform the data analysis.

This study and the data collection process took place in participants’ homes. Data were collected with a Google Home Mini smart speaker, which was provided to participants for research purposes. Each participant created a Google Assistant profile and a verbal reminder for taking their pain medication according to the prescribed schedule. Participants were trained on how to use the MEMS cap. First, the participants printed the name of their scheduled medication and placed their pills in the medication bottle. Afterward, they placed the MEMS cap onto the bottle. The participants were encouraged to place the MEMS bottles in the same location as where their routine medications were placed. Mints were placed in medication bottles instead of scheduled pain medications to remind participants to use medications in the MEMS cap bottle. Participants used the voice assistant and completed the reminder task for 4 weeks. After 4 weeks, participants logged onto their Google Home app profile to confirm the execution time of the voice assistant reminder. MEMS caps were collected after participants transferred their scheduled medications into the original bottles.

## Results

A total of 5 women aged 56 to 80 years self-reported chronic pain in multiple body locations, such as the back, joints, and extremities. The women self-reported having co-occurring chronic health conditions, including arthritis. The scheduled pain medications prescribed were meloxicam, naproxen, acetaminophen, a gabapentoid, and leflunomide (ie, a medication used to decrease rheumatoid arthritis inflammation). The average Brief Pain Inventory-Short Form scores ranged from 3 to 6, and pain interference scores ranged from 1 to 8. The average necessity of pain medication score was 15 (range 2-22), and the average score for concerns about using pain medication was 16 (range 6-22). The average PROMIS Self-Efficacy for Managing Symptoms Questionnaire T-score was 55.5 (range 48-64).

The scheduled medication dose times were 9 PM (Participant A), 9 PM (Participant B), 11 AM (Participant C), 10 PM (Participant D), 10 AM (Participant D), and 5:30 AM (Participant E). Adherence percentages ranged from 82% to 100%. The mean overall latency between the reminder deployment time and the MEMS cap opening time was 55 minutes (SD 100 minutes). The average absolute latencies varied among the 5 participants; the shortest average time was 17 minutes and the longest average time was 4.5 hours. Multimedia Appendix 1 shows a representation of the frequency of MEMS cap openings that occurred around the scheduled voice reminder time (0 minutes) for taking pain medication. After 168 voice assistant reminders, 25 (14.6%) resulted in the opening of MEMS caps within 5 minutes of the reminder, and 107 (63.7%) resulted in the opening of MEMS caps within 30 minutes of the reminder.

## Discussion

This feasibility study used medication event monitoring to measure medication implementation adherence to pain medications in older adults using voice assistant reminders. The preliminary findings suggest that the absolute latency in the time between voice assistant reminder deployment and scheduled pain medication use may be impacted by pain characteristics and beliefs about pain medications. The following discussion will elaborate on the results from this study’s data analysis.

The women in this study reported pain in multiple body sites. Self-reported pain intensity was mild to moderate, and pain interference ranged from mild to severe. These findings are consistent with those of published literature [1,2]. The women used scheduled analgesic medications for pain that are commonly prescribed for short-term use and were confident in managing their pain symptoms [3,12]. Unlike participants in prior studies, the participants in this study shared similar beliefs about the necessity of pain medications and concerns about pain medications [9,10,28]. Our participants' had low to high concerns about pain medications but generally believed that pain mediations were necessary for managing pain symptoms. Of the 5 women, only 1 had several major concerns about using their scheduled pain medications.

Our analysis of the data captured variable medication-taking behaviors in these 5 participants. Prior published evidence has demonstrated that pain interference and duration do not influence medication adherence [31]. Our preliminary results found that the participants were mostly adherent to pain medicines; their adherence percentages ranged from 82% to 100%. During the analysis of the implementation adherence to scheduled pain medications, we found that the latency time between voice reminder deployment and the opening of the MEMS cap varied among the participants [6]. One possible explanation for this may be found in the descriptive data we collected on each participant in the subsample. The women’s individual pain characteristics and beliefs may have influenced the timing of scheduled chronic pain medication use. A total of 4 women reported moderate pain intensity scores and had short latency times (in minutes). Those who reported moderate pain intensity and severe pain interference opened the MEMS cap before receiving the voice reminder. The participant that had the longest latency time (in minutes) in terms of opening the MEMS cap reported mild pain interference and had strong concerns about pain medications. These preliminary findings are worth additional exploration.

One criticism for using MEMS caps is that adherence results may be biased because users might start exhibiting strict medication-taking behaviors. Our 5 participants demonstrated a variety of patterns while using scheduled pain medications. As previously described, there were instances of the MEMS cap being opened before participants received the voice assistant reminder. Although we did not assess the rationale, earlier medication use could be attributed to moderate pain intensity, pain interference, the anticipation of the reminder, or an individual’s routine.

Older adults may not experience problems when taking an analgesic dose before the prescribed schedule, but the earlier use of such medications may increase the severity of their side effects in a person with decreased renal clearance. Aging can decrease the clearance of pain medications from the body and increase the risk of adverse drug events, especially in older adults undergoing polypharmacy [8]. If an older adult takes doses earlier, the medication’s side effects could temporarily reappear. This can contribute to poor medication adherence. Gabapentoids and opioids have potentially harmful side effects, such as dizziness and sedation, that usually go away after a period of time [3]. In contrast, waiting too long to take pain medication may decrease the effectiveness of the medication. Nonadherent behaviors related to pain medications, such as meloxicam and naproxen, can impact the kidneys and the gastrointestinal and cardiovascular systems. Skipping, abruptly stopping, or incorrectly taking gabapentoid doses can increase the risk of experiencing adverse symptoms [32]. Leflunomide is a medication that can impact liver enzyme levels and must be taken according to health care providers’ recommendations [33].

Although this study provided a glimpse into a subsample of individuals’ pain medication behavior patterns when given a reminder, there are several limitations. The subsample participants were female, and additional studies would need to explore latency times in male and female adults with pain. Further, we were not able to statistically analyze the relationships among the data on a small sample of participants. Future studies would need to observe a larger sample over several months and use a study design that compares variables with a control group.

Voice assistants for personal connectivity and entertainment are gaining popularity. This technology is readily available to help older adults with various tasks, such as setting medication reminders. Voice reminders may help cue patients to take medications, but the timing of medication use varies. Medication event monitoring systems or sensors can be used to track objective medication use behaviors, which can be used as health outcomes in mHealth interventions. The absolute time of medication use can be viewed as a part of medication implementation adherence to scheduled medications.

## Appendix

Multimedia Appendix 1 Frequencies of the MEMS cap being opened before and after the voice assistant reminder.

Multimedia Appendix 2 CONSORT-eHEALTH checklist (V 1.6.1).

## Abbreviations

mHealth: mobile health
PROMIS: Patient-Reported Outcomes Measurement Information System
