# Supplementary figures and images for: Voice Assistant Reminders and the Latency of Scheduled Medication Use in Older Adults With Pain: Descriptive Feasibility Study
Source: JMIR Form Res. 2021 Sep 28;5(9):e26361. doi: 10.2196/26361 (PMC8512193; doi:10.2196/26361)

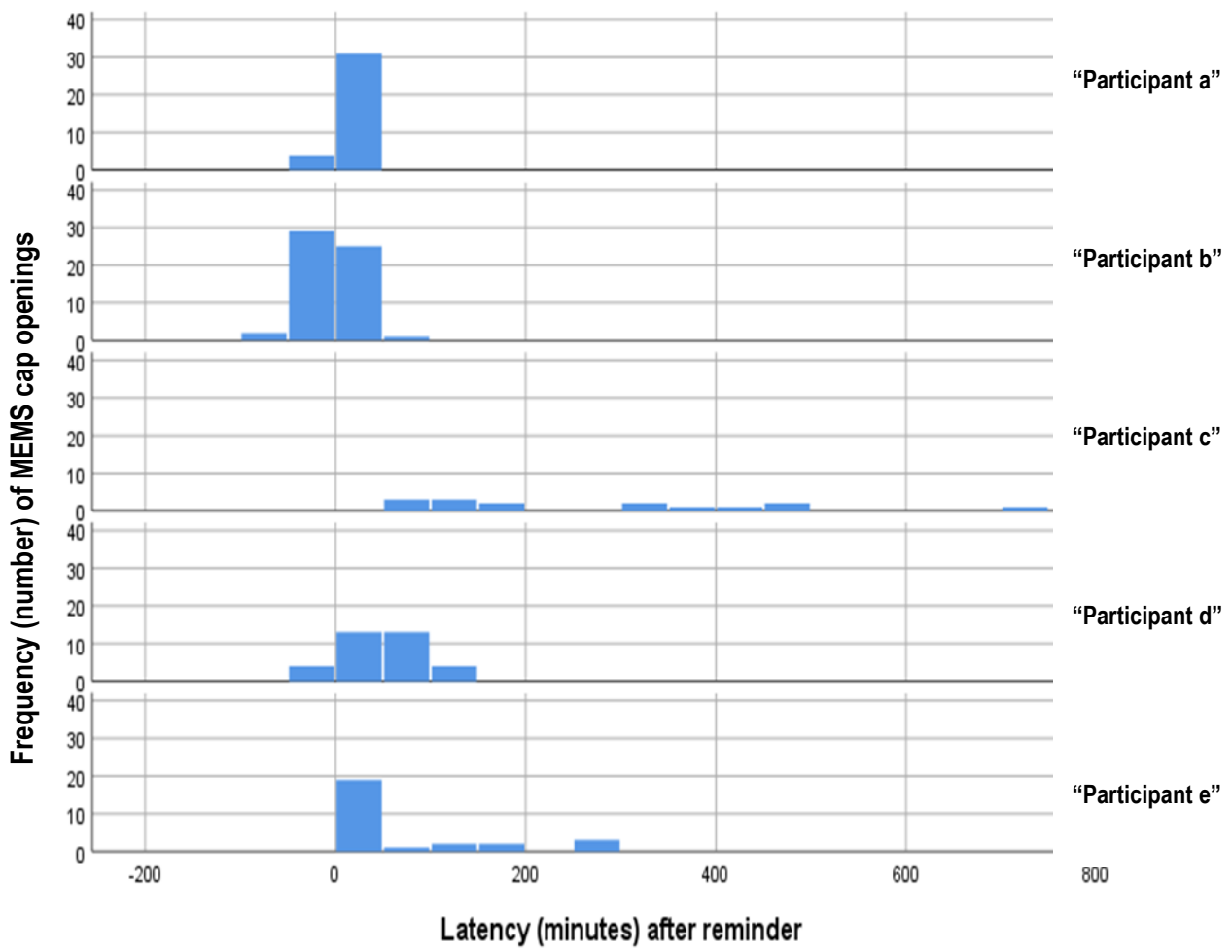

Supplement: Multimedia Appendix 1 [file formative_v5i9e26361_app1.pdf]
